# Improving Nursing Home Care through Feedback On PerfoRMance Data (INFORM): Protocol for a cluster-randomized trial

**DOI:** 10.1186/s13063-016-1748-8

**Published:** 2017-01-10

**Authors:** Matthias Hoben, Peter G. Norton, Liane R. Ginsburg, Ruth A. Anderson, Greta G. Cummings, Holly J. Lanham, Janet E. Squires, Deanne Taylor, Adrian S. Wagg, Carole A. Estabrooks

**Affiliations:** 1 Faculty of Nursing, University of Alberta, Edmonton, Alberta Canada; 2 Department of Family Medicine, University of Calgary, Cumming School of Medicine, Calgary, Alberta Canada; 3 Faculty of Health, York University, School of Health Policy and Management, Toronto, Ontario Canada; 4 School of Nursing, University of North Carolina at Chapel Hill, Chapel Hill, North Carolina USA; 5 Department of Medicine and Department of Family and Community Medicine, University of Texas, Health Science Center San Antonio, San Antonio, Texas USA; 6 School of Nursing, University of Ottawa, Ottawa, Ontario Canada; 7 Interior Health Authority, Kelowna, British Columbia Canada; 8 Faculty of Medicine and Dentistry, University of Alberta, Division of Geriatric Medicine, Edmonton, Alberta Canada; 9 Alberta Innovates-Health Solutions (AIHS) post-doctoral fellow, Translating Research in Elder Care (TREC), Faculty of Nursing, University of Alberta, 5-006 Edmonton Clinic Health Academy (ECHA), 11405 87 Avenue, Edmonton, AB T6G 1C9 Canada

**Keywords:** Audit and Feedback, Nursing Homes, Quality of Care, Quality Improvement, Performance, Cluster Randomized Controlled Trial, Clinical Microsystems

## Abstract

**Background:**

Audit and feedback is effective in improving the quality of care. However, methods and results of international studies are heterogeneous, and studies have been criticized for a lack of systematic use of theory. In TREC (*Translating Research in Elder Care*), a longitudinal health services research program, we collect comprehensive data from care providers and residents in Canadian nursing homes to improve quality of care and life of residents, and quality of worklife of caregivers. The study aims are to a) systematically feed back TREC research data to nursing home care units, and b) compare the effectiveness of three different theory-based feedback strategies in improving performance within care units.

**Methods:**

INFORM (Improving Nursing Home Care through Feedback On PerfoRMance Data) is a 3.5-year pragmatic, three-arm, parallel, cluster-randomized trial. We will randomize 67 Western Canadian nursing homes with 203 care units to the three study arms, a standard feedback strategy and two assisted and goal-directed feedback strategies. Interventions will target care unit managerial teams. They are based on theory and evidence related to audit and feedback, goal setting, complex adaptive systems, and empirical work on feeding back research results. The primary outcome is the increased number of formal interactions (e.g., resident rounds or family conferences) involving care aides – non-registered caregivers providing up to 80% of direct care. Secondary outcomes are a) other modifiable features of care unit context (improved feedback, social capital, slack time) b) care aides’ quality of worklife (improved psychological empowerment, job satisfaction), c) more use of best practices, and d) resident outcomes based on the Resident Assessment Instrument – Minimum Data Set 2.0. Outcomes will be assessed at baseline, immediately after the 12-month intervention period, and 18 months post intervention.

**Discussion:**

INFORM is the first study to systematically assess the effectiveness of different strategies to feed back research data to nursing home care units in order to improve their performance. Results of this study will enable development of a practical, sustainable, effective, and cost-effective feedback strategy for routine use by managers, policy makers and researchers. The results may also be generalizable to care settings other than nursing homes.

**Trial registration:**

ClinicalTrials.gov Identifier: NCT02695836. Date of registration: 24 February 2016

**Electronic supplementary material:**

The online version of this article (doi:10.1186/s13063-016-1748-8) contains supplementary material, which is available to authorized users.

## Background

### Importance of residential long term care

In Western countries, 3-8% of people aged 65 years or older live in nursing homes [1, 2] (e.g., 224 thousand in Canada [3], 1.3 million in the USA [4], and 2.9 million in Europe [1]), and demand for these services will substantially increase [1, 5, 6]. Between half and three quarters of nursing home residents have dementia (with a rising trend) [7–10], and these figures are likely underrated by at least 11% [11]. Dementia progresses from mild impairment and difficulties organizing daily life to incontinence, unsteadiness, profound difficulties in communication and nutrition intake, confinement to bed, and finally death [8, 12, 13]. In 2015, 46.8 million people aged 60 years or older were living with dementia worldwide (4.8 million in North America, and 7.5 million in Western Europe) [14], and 96% of US Americans with Alzheimer’s (the most common type of dementia) are 65 years or older [12]. Global numbers of older people with dementia will increase to 131.5 million by 2050. Currently, dementia has no prevention, cure, or effective treatment [12]. Without dramatic breakthroughs in either prevention or treatment, *as the number of frail elderly increases, so will their eventual need for nursing home care*.

As older adults with dementia are able to remain at home longer with community care, future trends will be for transition to a nursing home later in the dementia trajectory [5, 15]. This will further increase levels and complexity of care in these settings. At the same time, absolute staffing levels and proportions of regulated staff are low in nursing homes, and up to 80% of direct care is provided by unregulated care providers (care aides) with little or almost no formal training [16–19].

### Ongoing challenges to quality of care and use of best practices

Quality of care in nursing homes has been a challenge for decades [20–23] and evidence to support consistent quality improvement strategies is still lacking [21, 24]. Multiple international reports [23, 25–29] describe sub-optimal quality of care in nursing homes. For example, rates of adverse events (e.g., pressure ulcers) vary up to 10-fold across facilities [30], are much higher at particular times during a nursing home stay [30–34], and vary across nursing home ownership models [30, 35].

In acute and primary care, we know that persistent and deeply troubling research–practice gaps exist across countries, professions, and settings [36–39]. A reported 30–40% of patients do not receive evidence-based care and 25% receive unnecessary or potentially harmful care [40, 41]. In the nursing home sector these performance gaps result in deleterious resident outcomes (e.g., high modifiable symptom burden in the last year of life, unnecessary and inappropriate transfer to hospital especially in the last weeks of life) and deleterious workforce outcomes (e.g., high burnout, reduced job satisfaction). Almost no knowledge translation (KT) research has been undertaken in the nursing home sector [36, 42, 43] and only recently have there been calls for such research [44–46]. There is little work developing effective and efficient means by which to tailor and deliver ongoing performance data to foster intentional action to improve quality of care and worklife. There has been even less work rigorously evaluating such strategies. We located no work attempting to tailor *organizational context data* into performance data. Under these conditions, leaders in the nursing home sector are faced with daunting challenges in delivering acceptable standards of care. They have little guidance on how to feasibly identify actionable performance gaps over time or how to respond to them.

### The Translating Research in Elder Care (TREC) research program

This project is a key element of our long term program of research (*Translating Research in Elder Care – TREC*) focused on advancing KT science [47, 48]. TREC’s mission is to improve quality of care and quality of life for older adults in nursing homes and work life for their care providers. At its most fundamental level, the goal of KT in clinical settings is to move research (e.g., performance data) to action (e.g., performance improvement). TREC is an ongoing research program and aims to create actionable performance data to improve performance at the clinical microsystem (care unit) level in nursing homes. The microsystem has rarely been targeted before in performance improvement trials and possesses unique features that make it key to leading care interventions and driving change in these settings [49, 50]. Our previous research demonstrated that members of the clinical microsystem are the best potential target to drive positive change in this field [49, 50]. Findings from this investigation will add new insights on how actionable research data on modifiable elements of organizational context (framed as performance data) can be used to improve performance in a complex adaptive system such as a nursing home [51]. The next two sections describe the relevance of clinical microsystems and the microsystem work context for performance improvement.

### Relevance of clinical microsystems

Focusing on clinical microsystems as the target for improvement activities is central to our work [52–54]. These “small group[s] of people who work together on a regular basis to provide care to discrete subpopulations of patients” (p. 474) are essential building blocks of organizations and the health system [55], and a critical target level for patient safety interventions [56]. Care services are often organized at this microsystem level [57] and individual residents receive care on care units embedded in organizations [58]. Targeting improvement strategies to the microsystem level can potentially transform health care systems [55]. Emerging international evidence suggests that improvement efforts focusing on the microsystem level are successful [54, 59, 60]. We found that the microsystem level explained a significantly higher percentage of variance in work context outcomes (e.g., leadership, culture, evaluation) than did individual (resident/caregiver) or facility levels [49]. Using longitudinal data on three risk-adjusted quality indicators (QIs) from the Resident Assessment Instrument – Minimum Data Set (RAI-MDS) 2.0 [61], we found that facility level reporting masks important inter-unit/intra-facility variation; thus quality improvement interventions should target both facility and microsystem levels [50]. The intervention described here *targets the leaders of these clinical microsystems*. Using a complex adaptive system lens in the nursing home sector, Anderson [62, 63] suggested fostering manager development as the key to effective performance improvement. Similarly*,* McDaniel et al. [64–66] argue that health care organizations are complex adaptive systems.

### Relevance of the care unit work context

Studies in *nursing homes* have increasingly focused on the broad contextual influences of organizations. Generally, context is theorized as culture [67–71], where specific elements (e.g., person-centeredness, staff engagement) are identified, measured, and associated with outcomes of interest (e.g., quality of life or care). Reports associate more positive cultures (more person-centered, less controlling, more relationship-based) with lower rates of feeding tube placement [69], lower restraint use [72], reduction in anti-psychotic prescribing [73], and better quality of care [74]. The influence of context on successful adoption of innovation and on quality improvement success was suggested theoretically [75–85], examined empirically [57, 86–91], and has been the subject of several reviews [92–97]. The consensus is that context is an important influence on implementation success. Dopson and Fitzgerald [98] for example, synthesized 39 case studies probing context and found it an important mediator of innovation. They identified several contextual processes important to innovation adoption and, we argue, to using performance data to achieve performance goals. Among these processes were (1) sensing, interpreting, and integrating new evidence; (2) reinforcing or marginalizing new evidence; (3) relating new evidence to local context needs; and (4) discussing/debating new evidence with local stakeholders. Recent systematic reviews [96, 97, 99, 100] identified organizational features such as complexity, centralization, size, a research champion, organizational slack, and resources as important to innovation success. Positive influences include *cultural context* (culture, climate, openness to change, organizational innovativeness, leadership, evaluation, feedback), *structural context* (organizational structure, management and supervision, resources, time, staff development), *physical context* (organizational size), and *social context* (social influence, collaboration, relational capital, communication, participation in decision making). We have repeatedly demonstrated that organizational context as measured using the Alberta Context Tool (ACT) [77, 80, 101–105] has a positive association with better scores on staff outcomes (best practice use, burnout, job satisfaction) [57, 87, 88, 102, 103, 105] and recently have demonstrated this effect on resident outcomes [106].

### Purpose and aims

We seek to improve quality of care and performance/outcomes by translating findings on modifiable elements of organizational context (work environment). The purpose of this project is to systematically evaluate tailored interventions targeting the leaders of clinical microsystems in nursing homes. The interventions are designed to feed back performance data for improvement. Our aims are:

1. To evaluate and compare three feedback strategies – a standard feedback strategy and two assisted and goal-directed feedback strategies
2. To assess possible longer term effects of each strategy
3. To refine, based on the outcomes of this evaluation, a practical assisted feedback strategy for use in the nursing home sector that targets the leaders of their clinical microsystems

## Methods/Design

### Design

This is a protocol for a pragmatic, three-arm, parallel, cluster-randomized trial using stratified permuted block randomization with baseline assessment, a one-year intervention period, post-intervention assessment, and 18-months long-term follow-up (Table 1). This protocol followed the SPIRIT reporting guidelines for trial protocols [107] (Additional file 1). Should we need to make important modifications to this protocol, we will revise our study registration and ethics application (including all relevant study documents) accordingly, inform all relevant stakeholders (investigators, trial participants, regulators), and will report protocol changes in our final publication of study results. We have established a structure of committees and working groups to facilitate intervention development and implementation, and to ensure scientific rigor (Additional file 2). We will disseminate study results in peer-reviewed publications and international conference presentations.

**Table 1:** Schedule of enrolment, interventions, and assessments (adapted SPIRIT flow diagram [107])

	T_-2_ (09/14-04/15)	T_-1_ (10/15-11/15)	T_0_ (11/15-05/16)	T_1_ (05/16-06/16)	T_1_ (10/16-11/16)	T_1_ (04/17-05/17)	T_x_ (05/17-12/17)	T_x_ (01/19-06/19)
	Baseline	Dissemination workshop	Study preparation	Goal setting workshop	Support workshop 1	Support workshop 2	Post intervention	Long-term follow-up
Assessment of primary and secondary study outcomes *(all facilities participating in TREC)*								
TREC survey	X						X	X
RAI-MDS 2.0	X						X	X
Unit profile survey	X						X	X
Facility profile survey	X						X	X
Enrolment *(all TREC facilities in Alberta and British Columbia)*								
Ethics/operational approvals			X					
Eligibility screening			X					
Randomization/allocation			X					
Recruitment and informed consent *(BAF/EAF)*			X					
Study intervention								
Standard Feedback *(SF)*		X						
Basic Assisted Feedback *(BAF)*		X		X	X (virtual)	X (virtual)		
Enhanced Assisted Feedback *(EAF)*		X		X	X (face-to-face)	X (face-to-face)		
Ongoing phone/e-mail support *(EAF)*				–––––––	–––––––	–––––––		
Process evaluation								
Workshop evaluation surveys (at the end of each workshop)		X		X	X	X		
Workshop fidelity checklist and debriefs (at the end of each workshop)		X		X	X	X		
Detailed documentation of workshops (participant observations)					X (during workshops)			
Focus groups with *BAF/EAF* unit managers						X (05/2017)		X (12/2018)
Semi-structured interviews with *SF* unit managers					X (01/2017)			
Basic cost evaluation of the intervention				–––––––	–––––––	–––––––		

*BAF* Basic Assisted Feedback; *EAF* Enhanced Assisted Feedback; *SF* Standard Feedback

INFORM is set in urban nursing homes participating in our current phase of TREC – a stratified (region by size by operator) random sample of 91 urban sites. We recruited these sites recruited from three provinces: Alberta, British Columbia, Manitoba. These TREC facilities participate in a longitudinal observational study that generates a rich set of resident, staff, unit, and facility level measures and outcomes. Data from this ongoing observational study forms the baseline assessment, and data from future waves of this study will form the post-intervention and long-term follow-up assessments. We will invite all eligible sites in Alberta and British Columbia to participate in INFORM (sample information sheet and informed consent, Additional file 3). TREC Manitoba facilities are not eligible to INFORM, as they are participating in another TREC intervention study.

### Intervention

The following theoretical foundations informed intervention development: (a) audit and feedback in the health care literature, (b) feedback in the organizational literature, (c) goal setting theory, and (d) experiences from feedback activities during the first phase of our work (Additional file 4). The intervention target is the clinical microsystem *managerial team* within nursing homes: unit care managers and the director of care. We will feed back data about four aspects of organizational context that we routinely measure in our program with the validated ACT [80, 104]: (1) the number of *formal interactions* care aides have with other providers and with patients/families; (2) the amount of *slack time* care aides have; (3) *evaluation* (unit feedback) practices, and (4) *social capital*. We purposefully selected these four concepts and defined *formal interactions* (FI) as our primary outcome for the following reasons (details Additional file 5):

- of the ten ACT concepts, FI best reflects if a care unit has a more or less favorable work context, suggesting that improving FI improves context in general;
- FI scores are generally low in our study sample (mean = 1.32, possible maximum = 4), suggesting substantial room for improvement;
- based on theory and care unit managers’ opinions, FI is highly modifiable;
- systematically involving care aides in FIs (e.g., resident rounds) can provide care aides as well as the care team with crucial information about residents.

**Fig. 1:**
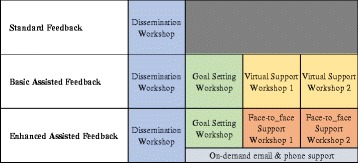
Intervention elements of the three study arms

The study will include three study arms: (1) Standard Feedback (SF), (2) Basic Assisted Feedback (BAF), and (3) Enhanced Assisted Feedback (EAF) (Fig. 1).

#### Dissemination workshops

All TREC sites have received SF in October/November 2015, which included participation in a face-to-face Dissemination Workshop. During these half-day workshops, we presented feedback reports with a particular focus on the core set of actionable context targets (FI, evaluation, social capital, slack time). A trained facilitator ran these workshops, a senior researcher presented on the reports, and participants engaged in small group discussions to: (a) help with interpretation of results overall, (b) draw attention to elements of context that are modifiable, (c) encourage microsystem teams to improve more modifiable context elements. We did not set specific goals – we provided simple “do your best” instructions.

#### Goal Setting Workshops

Facilities in the BAF and EAF arm will participate in an additional face-to-face Goal Setting Workshop. In each of four regions (Edmonton, Calgary, Interior Health, Fraser Health) we will hold a separate workshop for BAF and EAF sites. We expect an average number of 10-20 participants at each workshop (i.e., approximately 0.5-1 person per care unit). Care managers (who are the primary target group of this intervention) often oversee more than one care unit in their facility at the same time. We will prepare a Goal Setting Workshop Package for each care unit, including (a) a feedback report on the care unit’s context data (FI, evaluation, social capital, slack time), and (b) a Goal Setting Workbook summarizing important details on the INFORM study, defining key concepts, and outlining the goal setting approach we are going to apply. We will send this Goal Setting Workshop Package to each participating unit one week ahead of the workshop. The workshops will take place at a conference venue (e.g., a hotel) located in the respective health region of the participating facilities. The workshops will involve small group activities that adhere to feedback and goal setting approaches including: (a) reflecting on context data, (b) performance goal setting, including establishing a series of proximal and/or learning goals that will provide teams with markers of progress and explicit strategies for attaining performance goals, respectively, and (c) identification of ways care unit managers can gather interim feedback to assess progress towards goal achievement. The same trained facilitator as in the Dissemination Workshops will lead the Goal Setting Workshops. A researcher and regional decision maker dyad will be present with expertise in performance data and the specific clinical care setting, respectively. The same researcher will attend all workshops; the regional decision maker will be specific to each respective region. Participants will generate an action plan and will receive instructions for tracking goal progress and reporting at the support workshops. Additionally, we will assist workshop participants to develop specific, measurable goals and tools to track goal achievement. For example, if a care unit managerial team defines the goal to include at least four care aides in their monthly family conferences, we will provide managers with a run chart template to track the number of care aides included in each of those meetings during the study.

#### Support workshops

Six months after the Goal Setting Workshop we will hold a 90-min. virtual support workshop (webinars) in the BAF arm and a 180-min. face-to-face support workshop in the EAF arm (using the same conference venues as for the Goal Setting Workshops). Groups will (a) report on their progress with proximal/learning goals and strategies used toward performance goals, (b) discuss challenges they encountered, and (c) receive support from the researcher–decision maker dyad in addressing these challenges. BAF participants will receive limited online peer-to-peer support, EAF participants will benefit from face-to-face support from peers and research team members. Six months later we will hold a second support workshop in the BAF and EAF arms, similar in content to the first. The second support workshops are designed to continue to help participants discuss progress and problem solve. To ensure consistency in intervention delivery, the same trained facilitator will lead all BAF and EAF workshops, and the same researcher–decision maker dyad as in the Goal Setting Workshops will be present. Participants will receive push emails three months before each support workshop to remind them of the dates and the tasks to prepare.

#### On-demand e-mail and phone support

EAF teams will also have access to on-demand e-mail and phone support from the facilitator throughout the intervention period to address questions they may have and help resolve challenges that arise as they work towards goal achievement.

### Sample

#### Sample size calculation

The intervention target are the nursing home care units (clinical microsystems). To avoid contamination effects, we will randomize at the facility level, with all unit managers of the same facility receiving the same feedback intervention. Due to the three-arm design, multiple repeated measures, and the complex nested structure (time points nested within each care unit, and units clustered within facilities), basic methods of sample size estimation are not applicable [108–110]. Therefore, we adapted a computer simulation-based approach described by Arnold et al. [108] using the statistics software R (version 3.1.2) [111]. Power and sample size were based on the following mixed-effects regression model:

$$ {Y}_{ijt}=\mu +{\beta}_1\;A{1}_{ijt}+{\beta}_2\;A{2}_{ijt}+{\beta}_3\;A{3}_{ijt}+{b}_i+{b_i}_j+{\varepsilon}_{ijt} $$

- Y_ijt_ is the ACT *Formal Interactions* (FI) score (primary outcome) of unit j in facility i at time t.
- μ is the FI population mean.
- A1_ijt_, A2_ijt_, and A3_ijt_ are indicator variables for the interventions (SF, BAF, and EAF, respectively). The indicator variable is 1 if the unit has been exposed to the respective intervention and 0 otherwise.
- β_1_, β_2_, and β_3_ are the treatment effects of the three interventions.
- b_i_ is a facility-level random effect (variability of units within the facility).
- b_ij_ is a unit-level random effect (variability of time points within the unit).
- ε_ijt_ is a residual term (variability between units).

We assumed that the random effects and the residual term were normally distributed with mean zero and uncorrelated with one another. Using data from the previous phase of TREC (2007–2012) we estimated the following parameters to be entered into the model:

- μ = 1.1 (FI mean was 1.32. Standard feedback here is similar to that provided in the previous TREC phase [112–114]. We assumed that this intervention will, at least, have a small effect (increase the FI score by 0.2) compared to no feedback.
- Standard deviation of b_i_ = 0.154 (ICC = 0.445) (FI variability of units, within facilities)
- Standard deviation of b_ij_ = 0.104 (FI variability of units, across waves 1 and 2 in TREC 1.0)
- Standard deviation of ε_ijt_ = 0.192 (variability of the unit FI residual term)
- Average cluster size = 3 units per facility

**Fig. 2:**
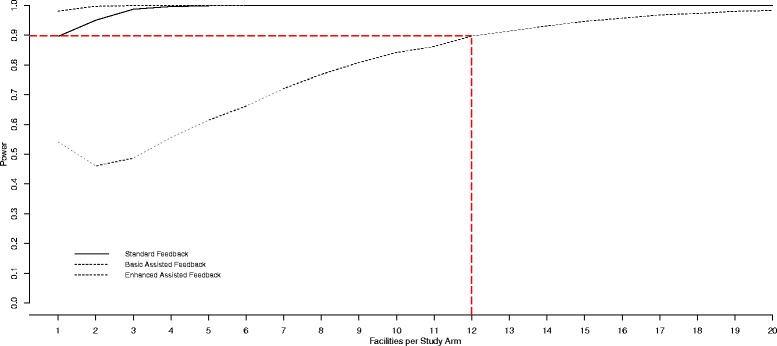
Results of the sample size calculations

We assumed that the FI score will increase by β_1_ = 0.2 in the SF group, by β_2_ = 0.4 in the BAF group, and by β_3_ = 0.6 in the EAF group. Based on these simulations (Fig. 2) 12 facilities per study arm (on average 3 units per facility) are required to detect the assumed effects with a statistical power of 0.90. To allow for attrition and effects smaller than the assumed ones, we will invite all eligible units in the 67 eligible facilities (see below) in Alberta and British Columbia for participation.

#### Inclusion and exclusion criteria

To be eligible (Table 2), facilities and units have to participate in the TREC observational study, as *INFORM* outcomes are only available for these facilities. We will only include facilities and units located in Alberta or British Columbia, as the TREC Manitoba facilities are participating in another TREC intervention study. Facilities need to have at least one unit with ten or more care aide responses on the TREC survey used to assess organizational context and staff outcomes. Only care units with ten or more care aide responses on the baseline TREC survey are eligible. From our previous work [115] we know, this number is required to ensure stable, valid and reliable aggregation of the study outcomes at the unit-level. From our observational study we know, there are two facilities for which we cannot assign HCA surveys to the microsystem. We excluded them as unit-level analyses are not possible for those facilities. We will only include units with an identifiable care manager or leader accountable.

**Table 2:** Inclusion and exclusion criteria for facilities and units

	Inclusion criteria	Exclusion criteria
Facilities	• Participation in the TREC observational study • Located in one of four health regions in Alberta (Edmonton or Calgary) or British Columbia (Fraser Health or Interior Health) • Have at least one care unit with ten or more care aide responses to our survey that we use to assess organizational context and staff outcomes at baseline (T_-2_)	• Does not participate in the TREC observational study • Located in the Winnipeg Regional Health Authority • Do not have at least one care unit with ten or more care aide responses to our survey that we use to assess organizational context and staff outcomes • Surveys collected in this facility cannot be assigned to a clinical microsystem as defined by TREC within this facility
Care units	• Located within a facility participating in the TREC observational study in Alberta or British Columbia • Ten or more care aide responses to our survey that we use to assess organizational context and staff outcomes are available • A care manager can be identified who leads this unit	• Not located within a facility participating in the TREC observational study in Alberta or British Columbia • Less than ten care aide responses to our survey that we use to assess organizational context and staff outcomes are available • No care manager can be identified who leads this unit

Based on these criteria, 203 units in 67 facilities are potentially eligible.

#### Enrolment, randomization, allocation

An independent person, not otherwise involved in this study, randomly assigned the 67 facilities to each of the three study arms. Randomization was stratified by health region (Edmonton, Calgary, Fraser Health, Interior Health) to account for the different policies within those regions that might influence organizational context and quality of care, and to facilitate delivery of the feedback intervention. We maintained regional proportionality of the 67 facilities within each study arm. From the list of facilities in each of the four regions, we selected the required number of facilities to be assigned to each of the three study arms by assigning a computer-generated random number to each facility. To facilities assigned to BAF or EAF we will offer *additional feedback*. We will explain to managers the specific extra feedback (treatment) they will receive, but we will blind them to group allocation.

#### Prevention of contamination

To limit the possibility of contamination, we will perform a cluster randomization. While we cannot prevent managers from talking to each other at regional or provincial meetings, contamination is less likely when study units are physically separate, interventions are more complex, and/or aim at changing behavior [116, 117]. We will also enlist participants’ agreement *a priori* to not share workshop tools with other managers or facilities during the study. The *INFORM* intervention is a complex intervention involving professionally facilitated face-to-face and virtual interaction with guided goal setting. It is therefore unlikely that managers in SF sites would be able to implement such an intervention unaided [118] or to change practice by virtue of interaction with intervention site managers at professional and other similar meetings. While we cannot fully blind all participants to all aspects of the study, we have undertaken comprehensive efforts to blind different stakeholder groups involved in INFORM as best as possible (details Additional file 6).

### Outcomes and instruments

Additional file 7 contains a summary table listing study outcomes, tools used to assess these outcomes, and their psychometric properties. Our outcome definition includes the name of the outcome, the time point each outcome will be assessed, the method of aggregation, the metric (e.g., change from baseline or group differences at a given time), the definition of the concept assessed, psychometric properties and an example item of each scale used.

#### Primary outcome

Based on arguments outlined in Additional file 5, we selected the ACT FI score as the primary outcome. FI is defined as “formal exchanges that occur between individuals working within an organization (unit) through scheduled activities that can promote the transfer of knowledge” [80]. The FI scale consists of four items asking care aides how often, in the last typical month, they participated in (a) team meetings about residents, (b) family conferences, (c) change-of-shift report, and (d) continuing education (conferences, courses) outside the nursing home (rated from 1 = never to 5 = almost always). The overall score is generated by recoding each item (1 and 2 to 0; 3 to 0.5; 4 and 5 to 1) and summing recoded values (possible range: 0–4).

#### Secondary outcomes

##### Organizational

We will assess three additional organizational context factors (evaluation, social capital, slack time) using the ACT, which is embedded within the TREC care aide survey, a suite of validated survey instruments completed by computer-assisted personal interview. We will also capture response to major near misses and managers’ organizational citizenship behavior, using data from our TREC unit survey, and performance reports and quality improvement activities, using data from our TREC facility survey. These instruments are described elsewhere [48, 49, 80, 102, 119].

##### Staff

We will capture instrumental and conceptual best practice use, psychological empowerment, job satisfaction, and a number of individual staff attributes, using the TREC care aide survey.

##### Residents

We will obtain resident data from the RAI-MDS 2.0, which is used internationally for comprehensive geriatric assessment of the health, physical, mental, and functional status of nursing home residents [61]. In Canada its use is mandated in several provinces/territories, as well as by the Canadian Institute of Health Information for national reporting [120]. Data for calculating QIs are captured in quarterly assessments completed on all residents [121]. The two practice sensitive (i.e., modifiable by care staff) QIs [122] worsening pain and declining behavioural symptoms will form the focus at unit and facility levels.

#### Process measures

##### Evaluation of the intervention sessions

We will adapt previously used questionnaires to the needs of this study. These surveys will include closed and open ended questions relating to participants’ intention to change, satisfaction with intervention components and with the intervention overall. These evaluation forms will be completed by participants at the end of the Goal Setting Workshops for both the BAF and the EAF arms, and at the end of the two support workshops; virtual for the BAF arm and face-to-face for the EAF arm.

##### Evaluation of intervention fidelity

To ensure that each intervention session is delivered as planned, consistently across arms and time, staff will work from study protocols. To evaluate protocol fidelity, study staff will observe, using a protocol checklist, adherence to workshop protocols during actual workshop delivery. There will also be space for staff to record field notes during each intervention session. We will track (a) how many of the participants invited to the workshops attended the workshops, (b) how many of the participants attended the workshop the full time, and (c) how many of the participants completed the workshop evaluations. For consistency of workshop delivery, the same facilitator and researcher will be present in all workshops. The regional lead will change for each region, but will be consistent across workshops within one region. For consistency of evaluation, the same staff member will complete field notes and fidelity checklist across workshops. This person will also run workshop debriefing sessions with the facilitator and researcher-regional decision maker dyad.

##### Evaluation of processes in the facilities

We will evaluate with the BAF and EAF managerial care teams: (1) to what extent they achieved the quality improvement goals defined in the workshops, (2) if they were able to apply the planned strategies in practice, (3) barriers and facilitators encountered, and (4) strategies applied to overcome challenges. We will conduct the evaluations as follows:

1. Detailed documentation of the first support workshop: We will document discussions and results in the first support workshop around the above named topics using detailed field notes, and we will analyze them using qualitative content analysis.
2. Focus Group 1: We will hold the first focus group one month following the second support workshop for BAF and EAF arms. The second support workshop is the final intervention component to which the participants are exposed. This will provide participants with the opportunity to reflect on the entire INFORM Intervention. We will ask participants in each region to sign up for a teleconference focus group session held at varying times to meet the needs of individual schedules (held separately for BAF and EAF groups).
3. Focus Group 2: We will hold the second focus group (organized the same way as the first focus group) one month before the long-term follow up data collection for both intervention arms. This will enable the research team to assess sustainability of the INFORM Interventions.

We will also conduct semi-structured interviews with SF care unit managers. We will ask questions about: people’s understanding of the ACT data, changes made on their unit as a result of feedback they received during the Fall 2015 TREC dissemination meetings, and barriers/facilitators to change. These interviews will allow us to compare the three study arms in terms of their ability to use the ACT data to improve context – the ultimate aim of INFORM. SF interviews will also provide information on additional quality improvement activities outside of INFORM, which may have contributed to unexpected “context” improvement during the intervention period.

##### Cost assessment

We will assess costs for *delivering* the INFORM interventions. We will not include costs related to *developing* the interventions and to *assessing the effectiveness* of the interventions (i.e., research costs). Using these data, we will assess and compare intervention delivery costs for the three study arms. We will collect data on all direct salary (workshop facilitator) and non-salary operating and travel expenses from University of Alberta accounting software, itemized by subcategory. The facilitator and study staff will keep detailed records of time invested in preparing and delivering each intervention. The cost of time invested by regional investigators and decision makers will be included. We will ask care unit managers monthly how much additional time and costs they and their staff spent on INFORM activities (i.e., not related to regular care or quality improvement activities), using standardized questions.

### Statistical analysis

#### Primary analysis

To compare the effectiveness of the three feedback strategies in improving the FI score, we will use *mixed-effects regression models*: multiple, linear, multi-level regression models including random and fixed (or mixed) effects.

#### Controlling for potential biases

We will account for multiple measures within each unit and clustering of units within facilities. We will adjust all analyses for the three stratification variables of the TREC facility sample (region, owner-operator model and facility size). We will compare characteristics of units and facilities using descriptive statistics at baseline, and adjust where there are significant differences between treatment groups (as baseline difference can occur by chance despite appropriate randomization). Should data not meet the assumptions of this model (multivariate normality, linearity, normally distributed, uncorrelated residuals, random effects with mean zero) the model will be adjusted accordingly. Within TREC, there is an extensive program to monitor and assure data quality, and a comprehensive data cleaning processes [123]. We will carry out an intention-to-treat analysis, as this best reflects the pragmatic nature of the study. We will compare these results to a per-protocol analysis, which better reflects adherence/non-adherence with the intervention. We will consider a care unit to be adherent with the intervention if at least one representative of this unit attends the Goal Setting Workshop and at least one of the two Support Workshops. The person attending the Goal Setting Workshop can be different from the person attending the Support Workshop(s). Units only attending the Goal Setting Workshop or not attending any of the workshops will be defined as non-adherent. We have registered the trial with ClinicalTrials.gov (NCT02695836) and we will use CONSORT guidelines [124] to report its findings.

#### Secondary analyses

We will monitor change of secondary outcomes over time in each study arm, and compare outcomes between three study arms using descriptive statistics, statistical process control methods and appropriate significance tests (t tests for normally distributed, linear, continuous outcomes; non-parametric tests for variables that do not meet these assumptions; chi-squared tests for categorical outcomes). We will longitudinally track two practice sensitive RAI QIs (worsening pain, declining behavioural symptoms), using statistical process control methods [125–128]. We will calculate risk-adjusted and unadjusted indicators and, using control charts, graphically display unit and facility performances. These data will enable us to assess possible effects of *INFORM* on resident care as measured by resident QIs at a time consistent with the intervention. We will assign a dichotomous variable (*improved/not improved*) to each unit in the intervention. Then, using logistic regression with *improvement* as the outcome we will investigate the effects of context (using ACT scales), best practice use, and staff characteristics on improvement. To this end we have developed a reliable classification system for individual control charts [49, 50].

## Discussion

INFORM is the first study to systematically assess effectiveness of different strategies to feed back research-based performance data to nursing home care units in order to improve their performance. In TREC we collect comprehensive longitudinal data on nursing home and care unit structural characteristics; modifiable features of nursing home and care unit work contexts; care providers’ use of best practices, health and quality of worklife; and various resident outcomes based on the RAI-MDS 2.0. Systematic feedback of these research data has been an important part of the long term TREC program. With INFORM we will be able to not only feed back data, but to systematically and comprehensively assess the most effective and cost-effective way to do so.

Our feedback interventions are based systematically on theory and evidence related to audit and feedback in the health care and organizational literature, goal setting, complex adaptive systems, and our own experiences with feeding back research results. This addresses Ivers and colleagues’ urgent call for systematic use of theory in future audit and feedback studies [129–131]. Furthermore, in order to determine which approaches to audit and feedback are most likely to change behaviours and improve performance, and to understand why these approaches work, head-to-head comparisons of different audit and feedback approaches, rather than comparison of audit and feedback with no intervention, are needed [129, 130].

We hypothesize that BAF and EAF will be significantly more effective than SF in improving formal interactions, context overall, and care provider as well as resident outcomes on nursing home care units. We furthermore expect that BAF and EAF will be similarly effective in doing so. However, we expect BAF to be more cost-effective than EAF. Results of this study will enable us to develop a practical, sustainable, effective, and cost-effective assisted feedback strategy that managers and policy makers can routinely use to improve performance of nursing home care units.

### Trial status

We have started recruitments of facilities and managerial teams on March 01, 2016, and recruitment is ongoing.

## Additional files

Additional file 1: INFORM_trial_protocol_add1_SPIRIT_checklist_30Apr2016.pdf, SPIRIT Checklist. (PDF 179 kb)
Additional file 2: INFORM_trial_protocol_add2_terms_of_reference_30Apr2016.pdf, Terms of reference INFORM committee and working group structure. (PDF 34 kb)
Additional file 3: INFORM_trial_protocol_add3_info_materials_30Apr2016.pdf, INFORM information sheet and informed consents. (PDF 57 kb)
Additional file 4: INFORM_trial_protocol_add4_theoretical_framing_30Apr2016.pdf, Theoretical framing of the study intervention. (PDF 180 kb)
Additional file 5: INFORM_trial_protocol_add5_primary_outcome_select_30Apr2016.pdf, Reasons for choosing Formal Interactions as primary study outcome. (PDF 188 kb)
Additional file 6: INFORM_trial_protocol_add6_blinding_30Apr2016.pdf, Blinding of INFORM stakeholders. (PDF 148 kb)
Additional file 7: INFORM_trial_protocol_add7_study_outcomes_30Apr2016.pdf, Primary and secondary study outcomes, and instruments used to assess these outcomes. (PDF 517 kb)
