# Supplementary material for: Improving Nursing Home Care through Feedback On PerfoRMance Data (INFORM): Protocol for a cluster-randomized trial
Source: Trials. 2017 Jan 10;18:9. doi: 10.1186/s13063-016-1748-8 (PMC5223357; doi:10.1186/s13063-016-1748-8)
Supplement: Additional file 2: — INFORM_trial_protocol_add2_terms_of_reference_30Apr2016.pdf, Terms of reference INFORM committee and working group structure. (PDF 34 kb) [file 13063_2016_1748_MOESM2_ESM.pdf]

Additional file 2: Terms of reference INFORM committee and working group structure

Committee	Role	Responsibilities	Accountable to	Team members	Meeting Details
INFORM Oversight Committee	Acts as final decision-making body for INFORM and provides overall direction to the project.<br><br>Resolves issues that arise from working groups	1. Oversees the overall implementation of the INFORM project<br>2. Reviews and approves protocols and policies<br>3. Addresses deviations from or modifications to trial and intervention protocols or their sub-protocols<br>4. Budget oversight<br>5. Monitors INFORM study deliverables	PI (Estabrooks) TREC Research Management Committee	Carole Estabrooks<br>Liane Ginsburg<br>Matthias Hoben<br>Ruth Anderson<br>Liane Ginsburg<br>Jayna Holroyd-Leduc<br>Janet Squires<br><br>Staff<br>Fiona Mackenzie<br>Daley Laing<br><br>Ex-officio<br>Cecilia Bukutu	Meets Monthly<br><br>teleconference<br><br>1 hour meetings

Committee	Role	Responsibilities	Accountable to	Team members	Meeting Details
Trial Working Group	Ensures research protocols are in place<br><br>Monitors the implementation of the research and intervention protocols assuring quality and fidelity	1. Develops the trial protocol and policies<br>2. Responsible for the Trial registration<br>3. Responsible for developing data collection and process measure tools<br>4. Works closely with the data unit to develop any feedback materials<br>5. Develops fidelity measures<br>6. Proposes publication and other dissemination activities	INFORM Oversight Committee	Carole Estabrooks<br>Peter Norton<br>Matthias Hoben<br>Liane Ginsburg<br><br>Staff<br>Fiona Mackenzie<br>Daley Laing<br><br>Ex-officio<br>Cecilia Bukutu	Weekly<br><br>teleconference<br><br>1 hour meetings

Committee	Role	7. Responsibilities	Accountable to	Team members	Meeting Details
Intervention Working Group	Develops and ensures delivery of the INFORM intervention	1. Operationalizes the project intervention plan and develops the intervention protocol<br>2. Develops the implementation strategy of the Goal Setting and the support workshops<br>3. Oversees (and develops) the development of intervention materials (workshop presentations, goal setting workbook)<br>8. Advises and makes recommendations to the Trail working group on any changes/deviations from the intervention protocol	INFORM Oversight Committee	Liane Ginsburg<br>Matthias Hoben<br>Don McLeod<br>Ruth Anderson<br>Dee Taylor<br><br>Staff<br>Fiona Mackenzie<br>Daley Laing<br>Alvina Ng<br><br>Ex-officio<br>Cecilia Bukutu	Weekly to bi-weekly as needed<br><br>teleconference<br><br>1 hour meetings
