# Supplementary material for: Improving Nursing Home Care through Feedback On PerfoRMance Data (INFORM): Protocol for a cluster-randomized trial
Source: Trials. 2017 Jan 10;18:9. doi: 10.1186/s13063-016-1748-8 (PMC5223357; doi:10.1186/s13063-016-1748-8)
Supplement: Additional file 3: — INFORM_trial_protocol_add3_info_materials_30Apr2016.pdf, INFORM information sheet and informed consents. (PDF 57 kb) [file 13063_2016_1748_MOESM3_ESM.pdf]

Additional File 3: INFORM information sheet and informed consents

Improving Nursing Home Care Through Feedback Of PerfoRMance Data (INFORM)

Information Sheet

Your facility is participating in a study entitled the *TREC Measurement System (TMS)*. In TMS we have provided participating facilities with feedback of the data we collected in TMS (i.e., the feedback reports you received and discussed in the regional summits in October 2015). Within the context of an additional study (INFORM), some TREC-TMS facilities have been selected to receive additional feedback. This information sheet provides information to enable you to make a decision about your participation. Please take the time to read this information carefully.

What is the purpose of the study?

In this study we will evaluate if the additional feedback package we are offering will be more effective at fostering improvements than the standard feedback package each TREC-TMS facility receives.

Why has my facility been invited to participate?

We have selected facilities that meet the following criteria:

- are participating in our TREC-TMS study
- are located in one of four health regions in Alberta (Edmonton or Calgary) or British Columbia (Fraser Health or Interior Health)
- have at least one care unit with 10 or more care aide responses to our survey that we use to assess organizational context and staff outcomes (the TMS survey)

Of all eligible facilities, we have randomly selected which ones will receive additional feedback interventions. Your facility has been selected by random for the additional feedback intervention.

What will taking part in the study involve?

The INFORM intervention will take part between March 2016 and the next wave of TREC-TMS data collection, which will start in July 2017. The number of units in you facility that will participate will depend on: (a) the number of units in your facility overall and (b) the number of units with 10 or more care aide responses to the TMS survey. Together, we will determine with you which units will participate. The target for our INFORM feedback intervention is the managerial teams (care managers and DoCs) of these selected units. In addition to the feedback report you received and to the regional summit you attended to discuss this report, you will receive the following additional feedback interventions:

- A **face-to-face Goal Setting Workshop** in May 2016, in which we will discuss your feedback reports in more detail, set specific performance goals for your participating units, and establish a series of learning goals that will provide your managerial teams with explicit strategies for attaining performance goals.
- Two **virtual (webinar-based) Support Workshops** in November 2016 and May 2017, in which managerial teams will (a) report on their progress in the proximal learning goals and strategies used toward their performance goals, (b) discuss challenges they may be encountering, and (c) receive support from the research team in addressing these challenges.
- **Semi-structured (qualitative, open ended) interviews** with participating managerial teams one month after each of the above mentioned workshops and 12 months after the INFORM intervention. During these interviews, we will evaluate (a) to what extent teams were able to

achieve the quality improvement goals defined in the workshops, (b) if teams were able to apply the planned strategies, (c) what barriers and facilitators teams encountered, and (d) which strategies teams applied to overcome challenges.

Participation in the study is voluntary. Members of the research team will provide detailed information on being involved in the study. Managerial teams will receive an information sheet similar to this one and will complete written informed consent before the semi-structured interviews.

What are the possible benefits of taking part in the study?

Participating in the study will help you improve quality of care on your care units, quality of work life of your care staff and quality of life of your residents and their family members. We will assist you to (a) identify specific areas for improvement, (b) define specific performance and learning goals, (c) develop effective strategies to achieve those goals, (d) identify and overcome barriers towards achieving those goals, and (e) identify and make effective use of facilitators of goal achievement.

How will we ensure confidentiality?

All information provided in interviews will be confidential. Information collected during this study will be linked to the unit and the facility but reports, publications, and presentations coming out of this study will not identify your unit or facility. The researchers and the decision-maker partners in your region will be aware of which facilities are participating in TREC.

What are the potential risks of taking part in the study?

There are no known risks to participating in this study.

Do I have to take part in the study?

Participation in any part of the study is voluntary and your facility can opt out at any time without penalty. Data collected up to the point of withdrawal from the study will be included in any data analyses. Your participation will not affect your employment at the facility.

Who is organizing the study?

The team running this project includes both university based researchers and knowledge users at the owner-operator, regional and ministry levels. This partnership approach ensures that the findings benefit the research community and have real-world applicability. The team includes a Principal Investigator who oversees the whole program of research and Regional Lead Investigators who will oversee the project in your region or zone. Details are provided below.

Project Role	Name & Title	Contact Details
Principal Investigator	Carole Estabrooks, Professor & Canada Chair (Knowledge Translation), Faculty of Nursing, University of Alberta	Carole.Estabrooks@ualberta.ca<br>(780) 492-3451
Alberta North Lead Investigator	Greta Cummings, Professor, Faculty of Nursing, University of Alberta	greta.cummings@ualberta.ca<br>780-492-8703
Alberta South Lead Investigator	Jayna Holroyd-Leduc, Associate Professor, Faculty of Medicine, University of Calgary	jmholroy@ucalgary.ca<br>(403) 944-1771
Fraser Health Lead Investigator	Jennifer Baumbusch, Assistant Professor, University of British Columbia,	jenniferbaumbusch@nursing.ubc.ca<br>(604) 822-7496

Interior Health Lead Investigator	Colin Reid, Assistant Professor, University of British Columbia	Colin.Reid@ubc.ca<br>250-807-9910

Contact Names and Telephone Numbers:
If you have any concerns about any aspects of this study, you may contact the INFORM Project Manager, Fiona MacKenzie (587) 987-3386. If you have concerns relating to research ethics, consent or otherwise, you may contact the Health Research Ethics Board of the University of Alberta at 780-492-2615. This office has no affiliation with the study investigators.

Thank you for taking the time to read this information sheet

Improving Nursing Home Care Through Feedback Of PerfoRMance Data (INFORM)

Informed Consent

Address

Date

Dear [insert name of Director of Care / Nursing Home Administrator],

Confirmation of participation in the Translating Research in Elder Care (TREC) project: Improving Nursing Home Care Through Feedback Of PerfoRMance Data (INFORM)

We would like to thank you and your facility for agreeing to participate in the above study.

Your signature on this form confirms your intention that [insert name of facility] will participate in the above named study and by doing so you agree on behalf of the facility to:

1) Have selected managerial teams participate in a regional face-to-face, half-day, Goal Setting Workshop in May 2016

2) Have selected managerial teams participate in two virtual (webinar-based) support workshops in November 2016 and May 2017

3) Allow a TREC team member to conduct semi-structured (qualitative, open ended) interviews with participating managerial teams one month after each of the above mentioned workshops and 12 months after the INFORM intervention

Contact Names and Telephone Numbers:
If you have any concerns about any aspects of this study, you may contact the INFORM Project Manager, Fiona MacKenzie (587) 987-3386. If you have concerns relating to research ethics, consent or otherwise, you may contact the Health Research Ethics Board of the University of Alberta at 780-492-2615. This office has no affiliation with the study investigators.

Thank you in advance for your time and assistance with this research study.

Yours sincerely,

Carole Estabooks, RN, PhD
TREC Principal Investigator

Professor & Canada Research Chair in Knowledge Translation
Faculty of Nursing, University of Alberta

Greta Cummings RN, PhD
Regional Lead Investigator

Professor, Faculty of Nursing
University of Alberta

Facility Administrator

Print Name ________________________________

Signature ________________________________

Facility Name ________________________________

Facility Address ________________________________

____

____
