# Supplementary material for: Improving Nursing Home Care through Feedback On PerfoRMance Data (INFORM): Protocol for a cluster-randomized trial
Source: Trials. 2017 Jan 10;18:9. doi: 10.1186/s13063-016-1748-8 (PMC5223357; doi:10.1186/s13063-016-1748-8)
Supplement: Additional file 4: — INFORM_trial_protocol_add4_theoretical_framing_30Apr2016.pdf, Theoretical framing of the study intervention. (PDF 180 kb) [file 13063_2016_1748_MOESM4_ESM.pdf]

Additional file 4: Theoretical framing of the study intervention

Audit and feedback in the health care literature

Audit and feedback (A&F) has received considerable attention in both the health care and the broader organizational literature as an approach to improving performance. Its overall impact has been low, the mechanisms leading to success are unclear [1-5], and its effectiveness is variable [2, 3, 6]. A recent systematic review of theory used to guide A&F RCTs targeting health professional practice or patient outcomes found that fewer than 10% of studies reported using theory in developing the A&F intervention [7]. However, this work was limited to A&F in the context of health care (with limited inclusion of nursing homes) and did not consider A&F studies other than RCTs. We located no systematic reviews on A&F in the nursing home sector and few rigorous studies have been done examining feedback strategies in this sector [8, 9]. In the health care literature, investigators have suggested that (1) use of A&F is a promising area of intervention but requires a more robust theoretical backing [2, 3, 7] and (2) further head-to-head comparisons are required to determine which approaches to A&F are most likely to lead to behaviour change and performance improvement [2, 3]. More specifically, Ivers et al. [3] argue that we need knowledge about the features of feedback that yield the best results in particular settings, specifically calling for attention to intrinsic features (method of delivery, content, timing) and extrinsic features (e.g., nature of the behaviour change, context of the target setting, policies). They found feedback to be most effective when it (1) was provided more than once, (2) was delivered in both verbal and written form, (3) included both explicit targets and an action plan, (4) addressed low baseline performance, (5) was delivered by a supervisor or senior colleague, and (6) aimed to decrease unwanted rather than to increase desirable behaviour. Therefore, in this study we will incorporate these features into our intervention.

Audit and feedback in the organizational literature

Work *external* to health care provides valuable insights into the role of feedback in motivating behaviour change and improving performance. At the individual level, this work is clear that the effectiveness of feedback is equivocal when it is used alone. However, results improve when feedback is used in conjunction with other approaches [10-12]. In particular, we recognize that *feedback* and *goal setting* are two of the most robust interventions available to improve learning and task performance [12]. The mechanisms by which feedback and goal setting work together to enhance performance involve other concepts, including self-efficacy and

attribution. Feedback operates as part of the self-regulatory processes set in motion when individuals accept a goal and work toward attaining it [13]. Personal standards set by individuals for themselves are translated into goals. These standards, along with feedback about their performance, have a motivational impact. When individuals work toward a goal, feedback provides them with information about the degree to which they attain that goal. Then, depending on their level of self-efficacy and the attributions they make, discrepancies between goals and performance either sustain motivation or cause them to abandon the task [13, 14]. As an example, a nurse manager sets a goal to demonstrate mastery of Pareto diagrams as part of a commitment to quality improvement. She completes exercises to test her grasp of the technique. If feedback shows that her performance is well below the goal set for herself, she may attribute failure to (1) lack of ability, which would lower her self-efficacy or cause her to abandon the task, or (2) lack of effort or poor instruction, which would not diminish self-efficacy, so task persistence would be the likely outcome. Goal attainment leads to enhanced self-efficacy and setting of higher performance goals [14].

A recent meta-analysis suggests that the goal setting–performance relationship holds at the group level [15]. DeShon et al. [16] found that the broader set of relationships between individual-level self-regulatory constructs (goal setting, feedback, self-efficacy, performance) function similarly at the group level. While some boundary conditions may not extend to use of learning goals on complex tasks at the group level [17], Kleingeld et al. [15] found that the goal setting–performance relationship at the group level held on both simple and complex tasks. Our study makes goal setting a core intervention component.

Goal setting and other key elements of a successful feedback intervention

Anseel et al. [18] recently tested a reflection strategy to stimulate deeper learning after feedback. They define reflection as a cognitive process in which people attempt to increase their awareness of personal experiences and therefore their ability to learn from them. They found that reflection combined with feedback enhances performance. As noted above, the impact of feedback alone on performance is unclear but feedback together with goal setting is one of the most robust interventions available to improve learning and task performance [12]. Assigned goals (the *tell and sell* method) can serve to enhance self-efficacy because a goal assigned by a respected authority appears attainable [11]. While Latham et al. [19] found that participation in goal setting enhanced performance, this was true only when *the process involved developing strategies to achieve the goal*.

The type of performance improvement targeted by *INFORM* has high task complexity [15]. Under conditions of high task complexity, Latham and Seijts [20] suggest establishing both proximal and distal goals. Proximal goals can be effectively used to break down the task and enhance self-efficacy and task persistence [11]. Learning goals added to performance goals are also found to be effective on complex tasks [21]. Providing people with learning goals (e.g., achieve mastery of Pareto charts) in addition to performance goals (e.g., decrease response time for lab results in an in-patient setting) on complex tasks is beneficial where knowledge of *how* to improve performance may be unclear. In these cases, learning goals can help generate a variety of task strategies to attain performance goals. Similarly, at both the individual level [10] and the group level [22], feedback is found to be particularly useful when outcome feedback (e.g., the extent to which a performance goal is achieved) is complemented by process feedback (e.g., appropriateness of the strategies used to attain the goal). At the group level Nahrgang and colleagues [17] found that, on complex tasks, learning goals can lead to more individualized focus among team members that hinders the task coordination necessary to enhance performance. However, other research on the group level [16] suggests that, as long as the intervention uses group-level feedback and group goals (not feedback and goals at the individual level), teams are able to allocate attention and motivation toward team performance without experiencing the deleterious effects noted by Nahrgang et al. [17]. Therefore, in this study we will focus on group goals and group feedback.

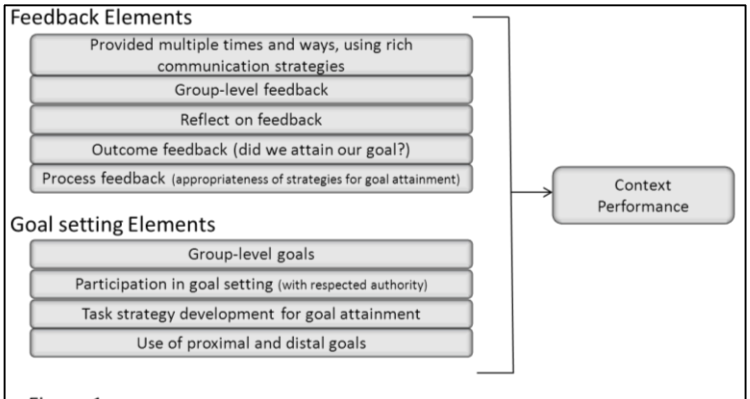

Figure 1

Finally, as noted earlier, the performance improvements targeted by *INFORM* take place in a complex environment where managers are faced with competing financial and clinical demands and priorities. We draw on work from the organizational literature [23] which suggests that, in instances where managers face conflicting interests, they need to use ‘rich’ information processing approaches (including face-to-face communication) to remove roadblocks. If feedback is to lead to performance improvement in complex health care environments, greater assistance in the feedback process is required, as suggested by a recent negative trial [24] of a feedback and goal setting intervention in primary care. In that trial, a minimally supported intervention had poor

compliance rates and did not lead to improvements in patient outcomes. Accordingly, we will use the key elements of a successful feedback intervention just outlined (Fig. 1), together with evidence from A&F work in health care described earlier and work on research dissemination,[25] in the design of our intervention.

Feedback activity during the first phase of our work

During the first phase (2007–2012) of the TREC program [26] we experimented with assessing, to a limited degree, the effects of providing research feedback to participants as the TREC program progressed [27-30]. We provided feedback through various *passive delivery methods* (posters, information sessions, regional meetings, facility annual reports) tailoring information to the particular target group. In addition to materials for front line workers (e.g., care aides, registered nurses, allied professionals), we produced materials for managers and administrators to illustrate (1) associations between more and less favourable organizational contexts, the use of best practices, and staff health, (2) unit and facility variation on modifiable elements of context, (3) staff health and well-being, and (4) resident outcomes. In this work we did *not* focus on performance goals or measure any effects of our activity beyond usual session evaluation (e.g., satisfaction). Here we aim to evaluate this *standard strategy* using learnings from our earlier work and to *compare it to two assisted strategies*.

References

1. Foy R, Eccles MP, Jamtvedt G, Young J, Grimshaw JM, Baker R. What do we know about how to do audit and feedback? Pitfalls in applying evidence from a systematic review. BMC Health Services Research. 2005;5:50.
2. Ivers N, Jamtvedt G, Flottorp S, Young JM, Odgaard-Jensen J, French SD, O'Brien MA, Johansen M, Grimshaw J, Oxman AD. Audit and feedback: effects on professional practice and healthcare outcomes. The Cochrane database of systematic reviews. 2012;6:CD000259.
3. Ivers NM, Sales A, Colquhoun H, Michie S, Foy R, Francis JJ, Grimshaw JM. No more 'business as usual' with audit and feedback interventions: towards an agenda for a reinvigorated intervention. Implementation Science. 2014;9:14.
4. Jamtvedt G, Young JM, Kristoffersen DT, O'Brien MA, Oxman AD. Does telling people what they have been doing change what they do? A systematic review of the effects of audit and feedback. Quality and Safety in Health Care. 2006;15:433-6.
5. Jamtvedt G, Young JM, Kristoffersen DT, O'Brien MA, Oxman AD. Audit and feedback: effects on professional practice and health care outcomes. Cochrane Database of Systematic Reviews. 2006:CD000259.
6. Kluger AN, DeNisi A. The effects of feedback interventions on performance: A historical review, a meta-analysis, and a preliminary feedback intervention theory. Psychol Bull. 1996;119:254-84.
7. Colquhoun H, Brehaut J, Sales A, Ivers N, Grimshaw J, Michie S, Carroll K, Chalifoux M, Eva K. A systematic review of the use of theory in randomized controlled trials of audit and feedback. Implementation Science. 2013;8:66.
8. Sales AE, Bostrom A-M, Bucknall T, Draper K, Fraser K, Schalm C, Warren S. The use of data for process and quality improvement in long term care and home care: A systematic review of the literature. Journal of the American Medical Directors Association. 2012;13:103-13.
9. Wagner C, van der Wal G, Groenewegen PP, de Bakker DH. The effectiveness of quality systems in nursing homes: a review. Quality in Health Care. 2001;10:211-7.
10. Earley PC, Northcraft GB, Lee C, Lituchy TR. Impact of Process and Outcome Feedback on the Relation of Goal Setting to Task-Performance. Acad Manage J. 1990;33:87-105.
11. Latham GP, Locke EA. Self-regulation through goal-setting. Organ Behav Hum Dec. 1991;50:212-47.
12. Locke EA, Latham GP. A theory of goal setting and task performance. Englewood Cliffs, NJ: Prentice Hall; 1990.
13. Bandura A, Cervone D. Self-evaluative and self-efficacy mechanisms governing the motivational effects of goal systems. J Pers Soc Psychol. 1983;45:1017-28.
14. Evans MG. Organizational behavior: the central role of motivation. J Manage Stud. 1986;12:203-22.
15. Kleingeld A, van Mierlo H, Arends L. The effect of goal setting on group performance: a meta-analysis. Journal of Applied Psychology. 2011;96:1289-304.
16. DeShon RP, Kozlowski SW, Schmidt AM, Milner KR, Wiechmann D. A multiple-goal, multilevel model of feedback effects on the regulation of individual and team performance. Journal of Applied Psychology. 2004;89:1035-56.
17. Nahrgang JD, Derue DS, Hollenbeck JR, Spitzmuller M, Jundt DK, Ilgen DR. Goal setting in teams: The impact of learning and performance goals on process and performance. Organ Behav Hum Decis Process. 2013;122:12-21.
18. Anseel F, Lievens F, Schollaert E. Reflection as a strategy to enhance task performance after feedback. Organ Behav Hum Decis Process. 2009;110:23-35.
19. Latham GP, Winters DC, Locke EA. Cognitive and motivational effects of participation - a mediator study. Journal of Organizational Behavior. 1994;15:49-63.
20. Latham GP, Seijts GH. The effects of proximal and distal goals on performance on a moderately complex task. Journal of Organizational Behavior. 1999;20:421-9.
21. Winters D, Latham GP. The effect of learning versus outcome goals on a simple versus a complex task. Group Organ Manage. 1996;21:236-50.
22. Hollenbeck JR, Ilgen DR, Lepine JA, Colquitt JA, Hedlund J. Extending the Multilevel Theory of Team Decision Making: Effects of Feedback and Experience in Hierarchical Teams. Acad Manage J. 1998;41:269-82.
23. Thomas JB, Trevino LK. Information-processing in strategic alliance building – a multiple-case approach. J Manage

Stud. 1993;30:779-814.

24. Ivers NM, Tu K, Young J, Francis JJ, Barnsley J, Shah BR, Upshur RE, Moineddin R, Grimshaw JM, Zwarenstein M. Feedback GAP: pragmatic, cluster-randomized trial of goal setting and action plans to increase the effectiveness of audit and feedback interventions in primary care. Implementation Science. 2013;8:142.

25. Huberman M. Research utilization: the state of the art. Knowledge and Policy. 1994;7:13-33.

26. Estabrooks CA, Squires JE, Cummings GG, Teare GF, Norton PG. Study protocol for the Translating Research in Elder Care (TREC): Building context – an organizational monitoring program in long-term care project (project one). Implementation Science. 2009;4:52.

27. Estabrooks CA, Teare G, Norton PG. Should we feed back research results in the midst of a study? Implementation Science. 2012;7:87.

28. Bostrom A-M, Cranley L, Hutchinson AM, Cummings GG, Norton P, Estabrooks CA. Nursing home administrators' perspectives on a study feedback report: A cross sectional survey. Implementation Science. 2012;7:88.

29. Hutchinson AM, Batra-Garga N, Cranley L, Bostrom A-M, Cummings GG, Norton P, Estabrooks CA. Feedback reporting of survey data to healthcare aides. Implementation Science. 2012;7:89.

30. Cranley L, Birdsell J, Norton PG, Morgan DG, Estabrooks CA. Insights into the impact and use of research results in a residential long-term care facility: A case study. Implementation Science. 2012;7:90.
