# Supplementary material for: Improving Nursing Home Care through Feedback On PerfoRMance Data (INFORM): Protocol for a cluster-randomized trial
Source: Trials. 2017 Jan 10;18:9. doi: 10.1186/s13063-016-1748-8 (PMC5223357; doi:10.1186/s13063-016-1748-8)
Supplement: Additional file 5: — INFORM_trial_protocol_add5_primary_outcome_select_30Apr2016.pdf, Reasons for choosing Formal Interactions as primary study outcome. (PDF 188 kb) [file 13063_2016_1748_MOESM5_ESM.pdf]

Additional file 5: Reasons for choosing Formal Interactions as primary study outcome

1. **Data-based argument.** Knowing that, as outlined in the background, organizational context is associated with staff and resident outcomes, we assessed which ACT concepts best explained the assignment of a care unit to either a high or low context group, compared to the allocation based on all 10 ACT concepts. With 74% agreement (Cohen's Kappa = 0.52, p<.0001), FI was the single ACT concept that correctly specified the highest proportion of care units as either high or low context. Of all combinations of ACT concepts, *Formal Interactions (FI)*, *Evaluation*, *Social Capital*, and *Organizational Slack–Time* demonstrated the highest agreement with the 10-concept analysis (Cohen's Kappa = 0.81, p<.0001). In addition, at the microsystem level in our earlier work, no unit scored FI values above 1.88 (of a possible maximum of 4) and the mean was 1.32, leaving substantial room for improvement in FI.
2. **Expert opinion-based argument.** We asked 16 care managers from three of our regional health authorities to assess the ten ACT concepts for potential to be feasibly changed. They ranked formal interactions along with evaluation and social capital as most modifiable, while slack time was considered less changeable and represents a 'stretch goal.'
3. **Theory-based argument.** The FI scale consists of four items asking care aides how often, in the last typical month, they participated in (a) team meetings about residents, (b) family conferences, (c) change-of-shift report, and (d) continuing education (conferences, courses) outside the nursing home (rated from 1=*never* to 5=*almost always*). Our 2007–2012 data indicated significant room for improvement across all sites. The *FI* score of a unit therefore can be improved by involving care aides more frequently in these encounters, an aspect of unit context that is easily modifiable. FI's also pose only modest additional burden to the system. Improving scores on other concepts could require investment in infrastructure modifications, hiring additional or more qualified personnel, or modifying organizational culture (which is difficult to modify) [1]. FI's make use of existing resources and require little investment beyond organizational adaptations (e.g., scheduling training or meeting sessions, developing educational materials to disseminate research findings). Importantly, FI is also a **proximal goal**. The organizational behaviour literature is clear that the goal set should not be too distal.

References

1. Shier V, Khodyakov D, Cohen LW, Zimmerman S, Saliba D. What does the evidence really say about culture change in nursing homes? Gerontologist. 2014;54:S6-S16.
