# Supplementary material for: Improving Nursing Home Care through Feedback On PerfoRMance Data (INFORM): Protocol for a cluster-randomized trial
Source: Trials. 2017 Jan 10;18:9. doi: 10.1186/s13063-016-1748-8 (PMC5223357; doi:10.1186/s13063-016-1748-8)
Supplement: Additional file 6: — INFORM_trial_protocol_add6_blinding_30Apr2016.pdf, Blinding of INFORM stakeholders. (PDF 148 kb) [file 13063_2016_1748_MOESM6_ESM.pdf]

Additional File 6: Blinding of INFORM stakeholders

The first principle in an RCT is to blind everyone possible to group allocation of sites – in order to reduce or eliminate introduction confounding factors that will bias the analyses and findings. With this in mind:

Randomization and study arm allocation

- Randomization of facilities was done by a TREC investigator not involved in any other way in the INFORM study.
- This investigator will keep and manage the master list of randomized facilities. He will be responsible for resolving any issues related to assignment of facilities during the recruitment process. He consults with the principal investigator in case of any major or non-resolvable issues. The TREC managing director (not involved in INFORM) keeps a back up copy of the master list.

Recruitment

- As RPCs are responsible for recruiting facilities in their respective region, they cannot be blinded to study arm allocation of facilities in their own region. Facilities in different study arms will get different recruitment materials (information sheets and informed consents), and RPCs need to know which information to pass along to the facilities.
- We cannot blind RPCs to allocation of facilities in the region they are responsible for, but we will blind each RPC for allocation of facilities *in all other regions*. RPCs cannot talk about allocation of their facilities to each other or to anyone else.
- The RPCs will **not** be involved in collecting the data for the post-intervention and long-term follow up outcome assessment in their region. If needed regional coordinators can help out with collecting outcome data in a region other than their own (i.e., in which they have been blinded to INFORM study arm allocation of sites).
- Facilities in the basic assisted feedback (BAF) and enhanced assisted feedback (EAF) arms will be informed that they are receiving additional feedback **without disclosing** details of the other arms of the intervention. Standard feedback facilities will not be informed that there are two additional study arms.

Intervention delivery

- The regional project coordinator (RPC) will enlist each facility's agreement ahead of time to not share workshop tools with other managers or facilities during the study.
- While we cannot prevent managers/directors of care from talking to each other at various regional or provincial meetings – the strategy above is often used in education research involving school. Additionally, with complex interventions such as this the kinds of conversations that may go on at meetings is unlikely to alter performance in any of the three arms.
- Nobody involved in the intervention delivery – facilitator, regional lead investigators, TREC trainees, other TREC investigators, TREC staff, etc. – can talk about facilities by name or share any information that can lead to identification of a facility or of its allocation status.

Fidelity monitoring

- The three investigators monitoring fidelity across all intervention arms, will limit their exposure to allocation by working only with files that have been assigned a random facility and unit identifier in an effort to remain completely blind to allocation.

Data analysis

Data analysts and TREC staff will be blinded as follows:

- The investigator holding the randomized master list of INFORM facilities will generate for each INFORM facility and care unit a unique random number that will be used as identifier. This identifier is different from the facilities' and units' TREC identifier in order to prevent analysts or any other study staff from identifying any of the facilities.
- The TREC managing director and one of the TREC research assistants will be the only additional persons, who have access to the de-identification list. Any of those three persons will keep these information confidential and will not share it with anybody else.
- RPCs or facilities will send materials (i.e., questionnaires, tracking sheets, etc.) directly to the TREC managing director. The TREC research assistant will then assign the unique INFORM identifier of the respective unit and facility to the materials, before anybody else can proceed with entering, analyzing, filing, etc. the materials.
- Should any person other than the managing director or research assistant by mistake receive INFORM materials sent in by the RPCs or facilities, this person has to pass the received mail immediately to the managing director, without opening it.
- Analyses of INFORM data will only be done with data that has been assigned the unique INFORM identifier.

Summary of blinding

INFORM Team Member	Level of blinding
**Principal Investigator, Carole Estabrooks**	Will be consulted in the case of any major or non-resolvable issues
**Co-investigator, Matthias Hoben**	Will monitor fidelity across all intervention arms<br>Work only with files and documents in which the unique TREC unit and facility identifiers have been replaced by the unique INFORM identifier
**Co-investigator, Liane Ginsburg**	
**Co-investigator, Peter Norton**	
**Independent Investigator, Malcolm Doupe**	Manager of the master list of randomized facilities<br>Will generate unique identifiers for INFORM facilities and care units<br>Access to de-identification list<br>Not involved in any other aspects of INFORM
**TREC Managing Director Cecilia Bukutu**	Holds back up copy of master list of randomized facilities<br>Access to de-identification list<br>All materials (from RPC's or facilities) sent directly to CB
**TMS Project Coordinator, Alvina Ng**	Access to de-identification list<br>Assigns unique INFORM identifier upon receipt
**INFORM Project Coordinator, Fiona Mackenzie**	Will not communicate any information related to allocation or facility identification to other regional coordinators, TREC staff, investigators, regional leads, decision makers, etc.<br>Work only with files and documents in which the unique TREC unit and facility identifiers have been replaced by the unique INFORM identifier
**INFORM Research Assistant, Daley Laing**	
**Regional Project Coordinators**	Aware of allocation of facilities in own region<br>Will not know allocation in any other region
**Regional Lead Investigators**	If necessary (assisting with recruitment or attending workshops), may know allocation of facilities in own region<br>Will not know allocation in any other region
**TREC data unit staff**	Work only with files and documents in which the unique TREC unit and facility identifiers have been replaced by the unique INFORM identifier
